# Supplementary figures and images for: CXXC-finger protein 1 associates with FOXP3 to stabilize homeostasis and suppressive functions of regulatory T cells
Source: eLife. 2025 Apr 4;13:RP103417. doi: 10.7554/eLife.103417 (PMC11970909; doi:10.7554/eLife.103417)

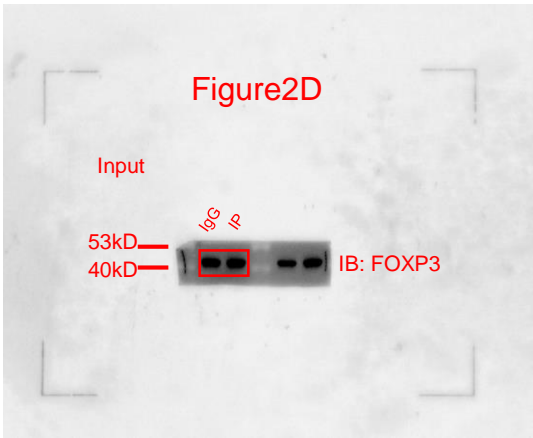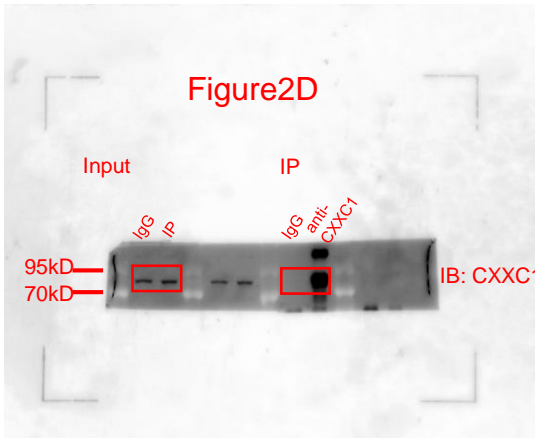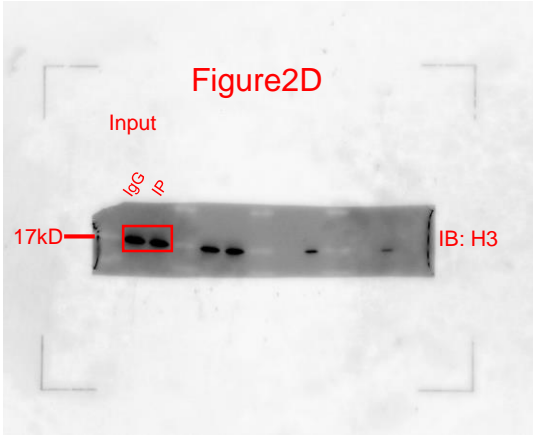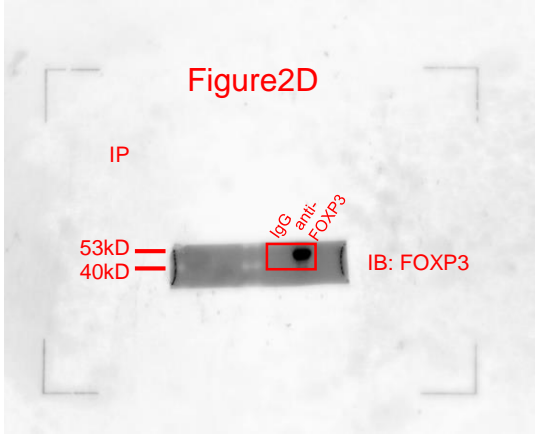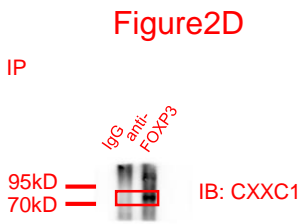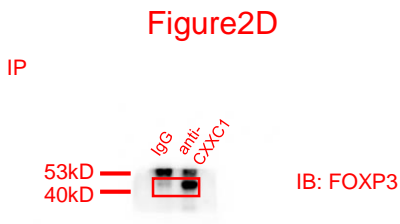

Supplement: Figure 2—source data 1. [file elife-103417-fig2-data1.zip › Figure 2-source data 1.pdf]

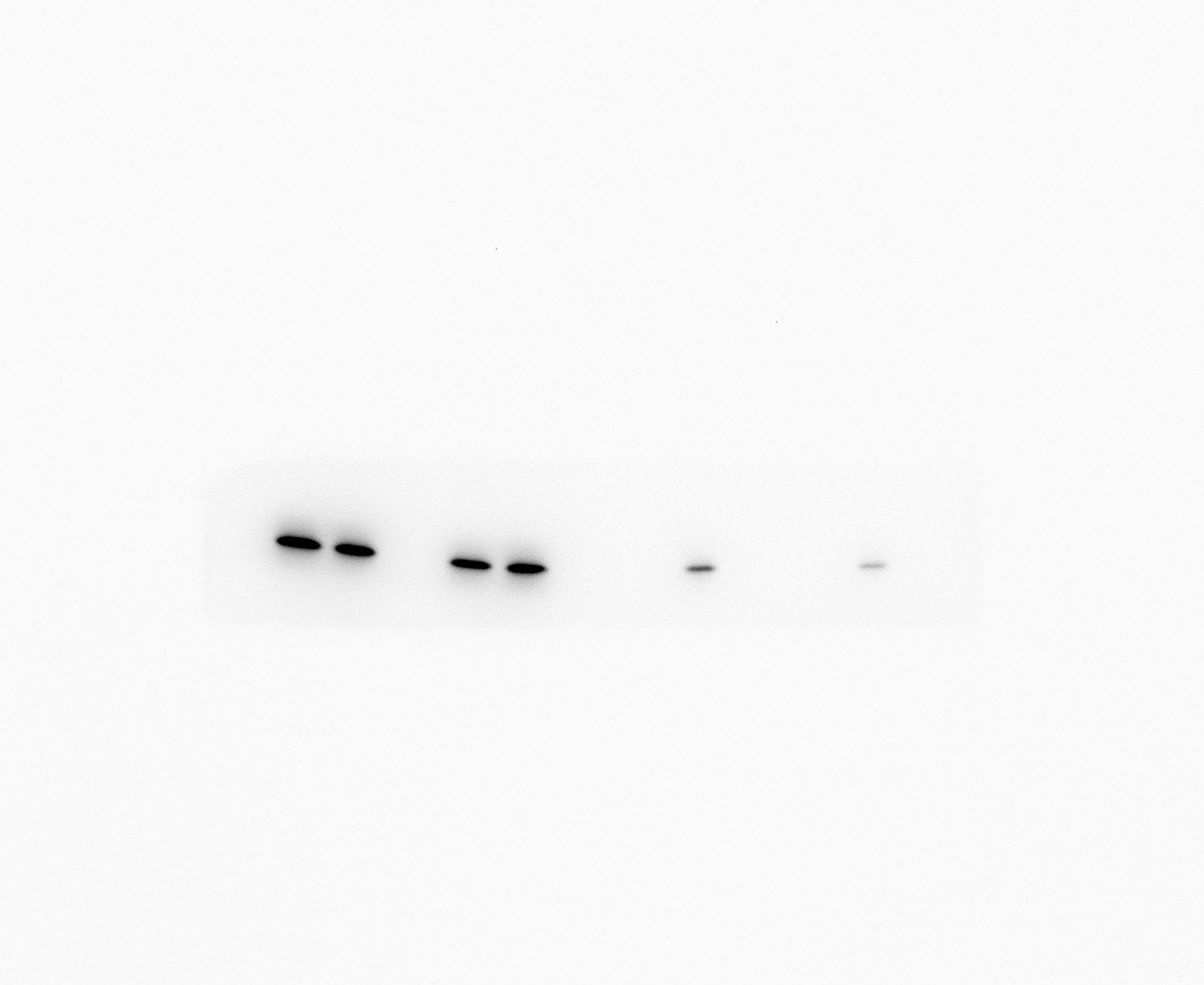

Supplement: Figure 2—source data 2. [file elife-103417-fig2-data2.zip › Figure 2-source data 2/H3.jpg]

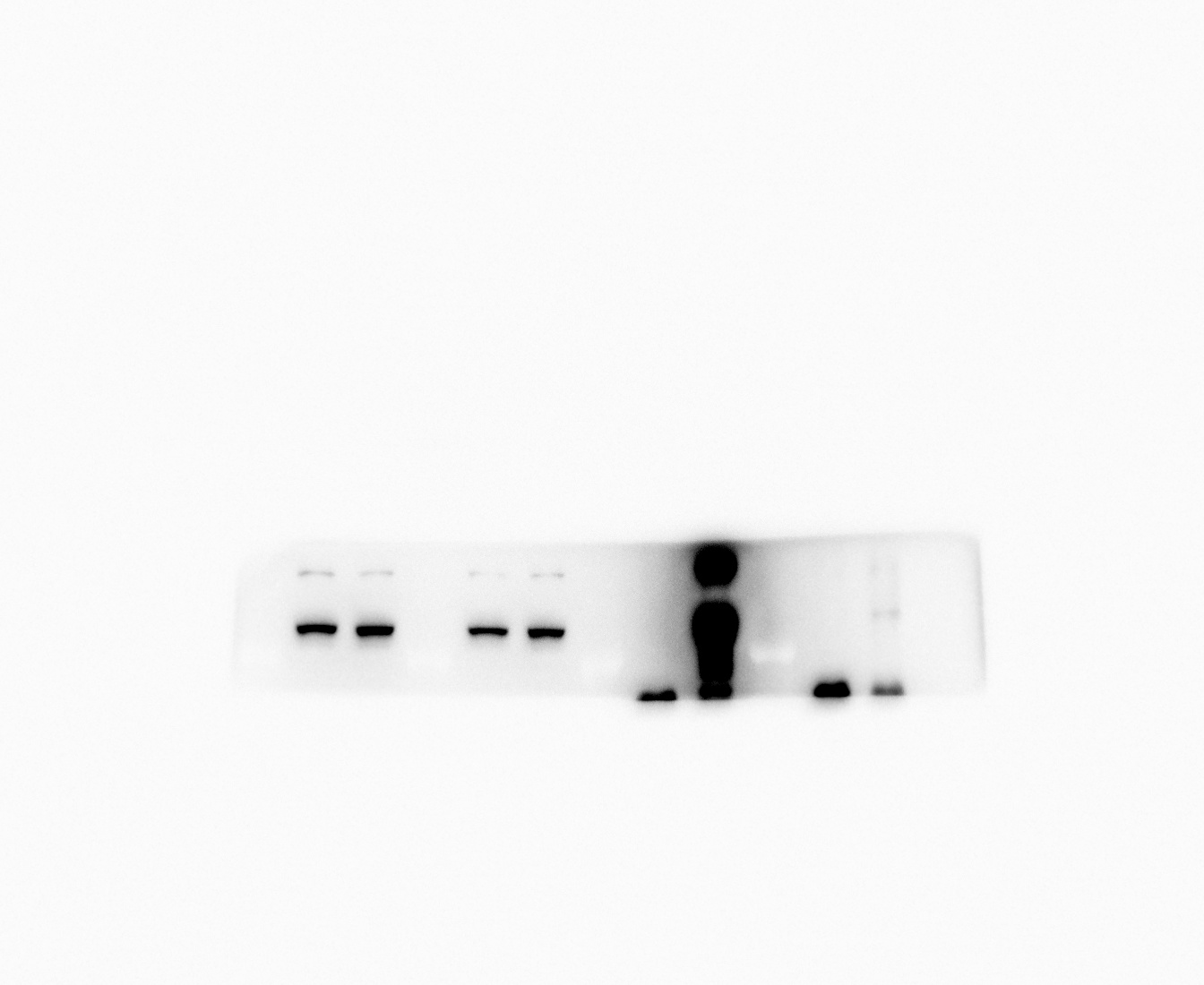

Supplement: Figure 2—source data 2. [file elife-103417-fig2-data2.zip › Figure 2-source data 2/Input IB CXXC1.jpg]

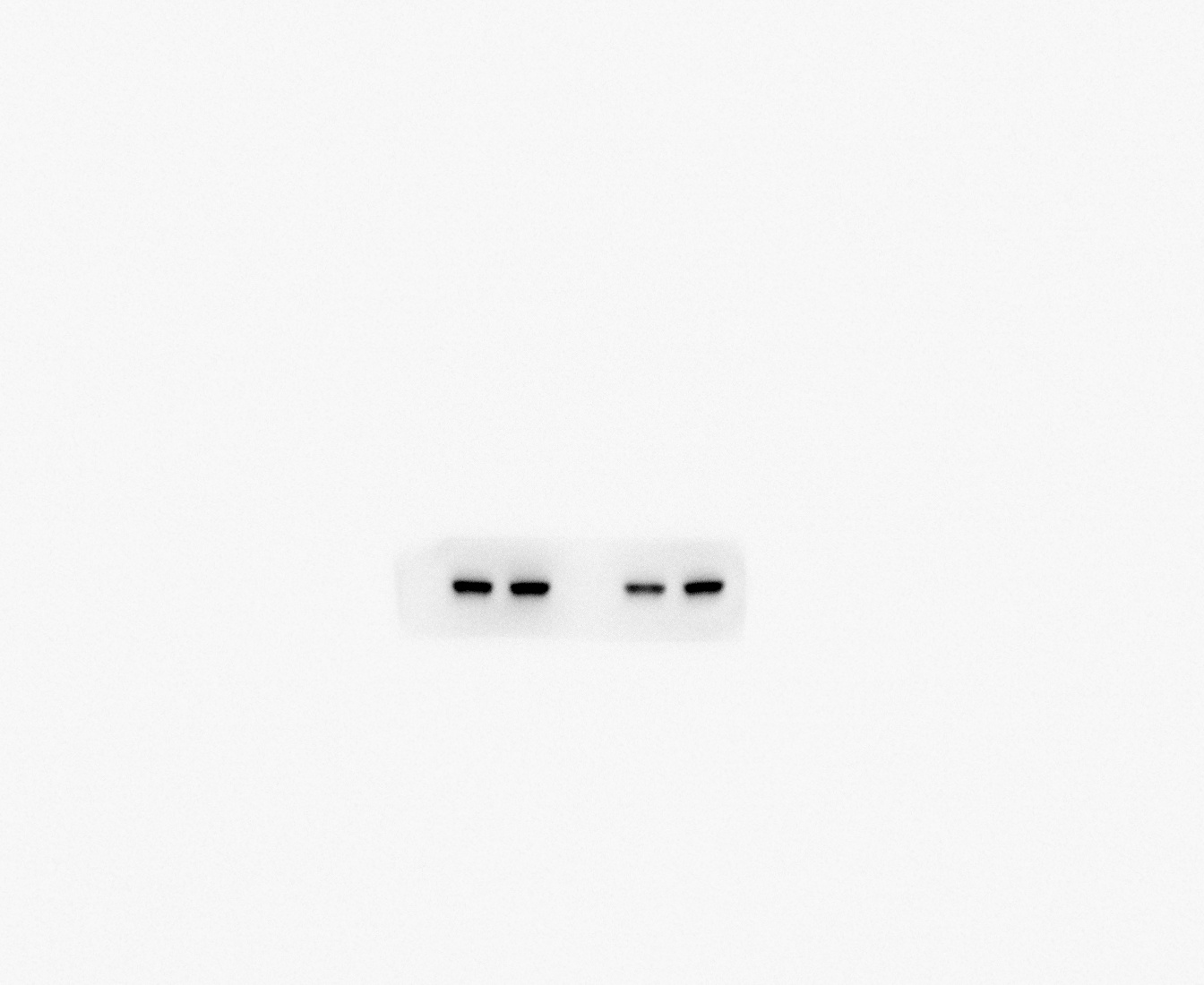

Supplement: Figure 2—source data 2. [file elife-103417-fig2-data2.zip › Figure 2-source data 2/Input IB FOXP3.jpg]

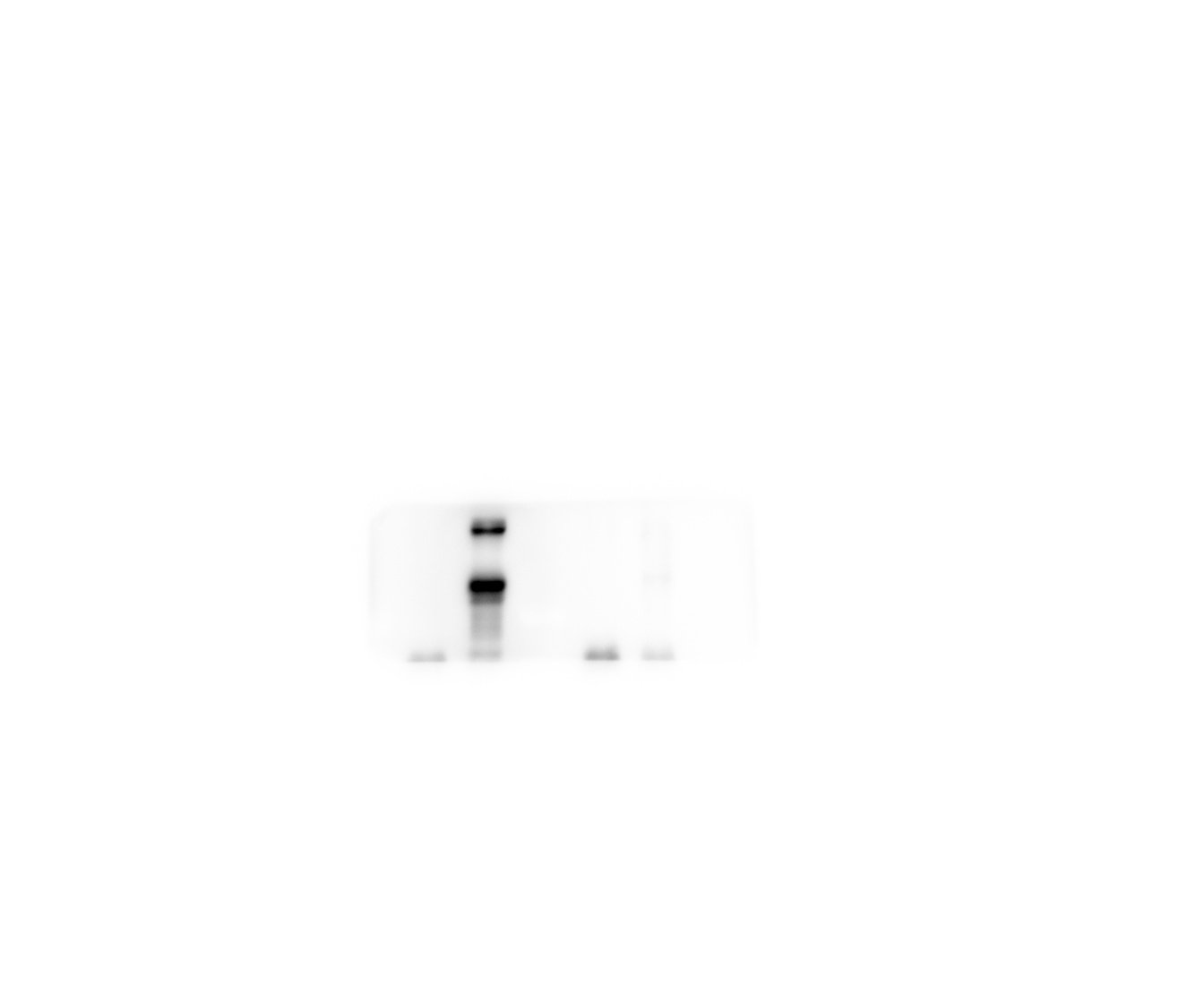

Supplement: Figure 2—source data 2. [file elife-103417-fig2-data2.zip › Figure 2-source data 2/IP-CXXC1 IB-CXXC1.jpg]

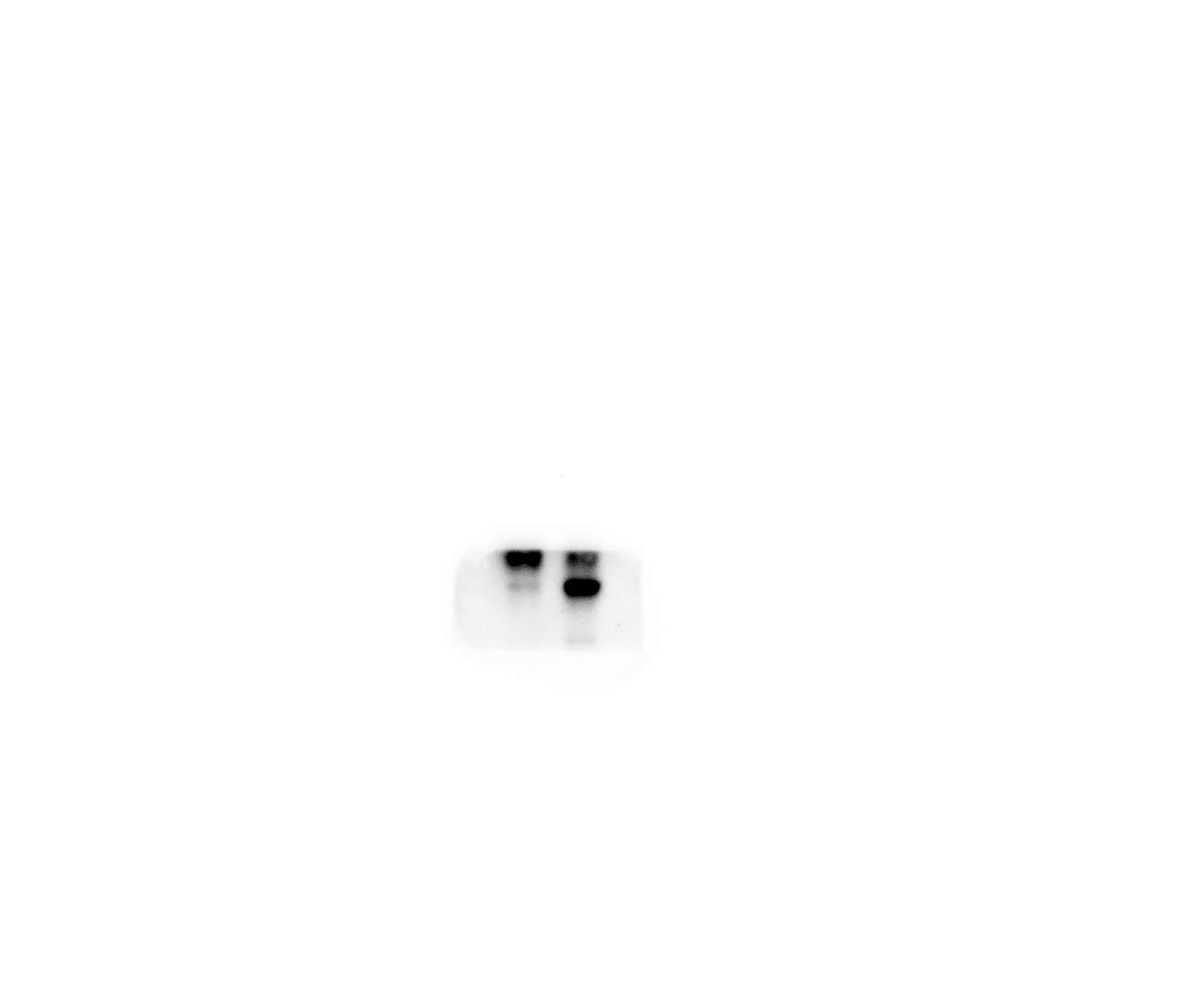

Supplement: Figure 2—source data 2. [file elife-103417-fig2-data2.zip › Figure 2-source data 2/IP-CXXC1 IB-FOXP3.jpg]

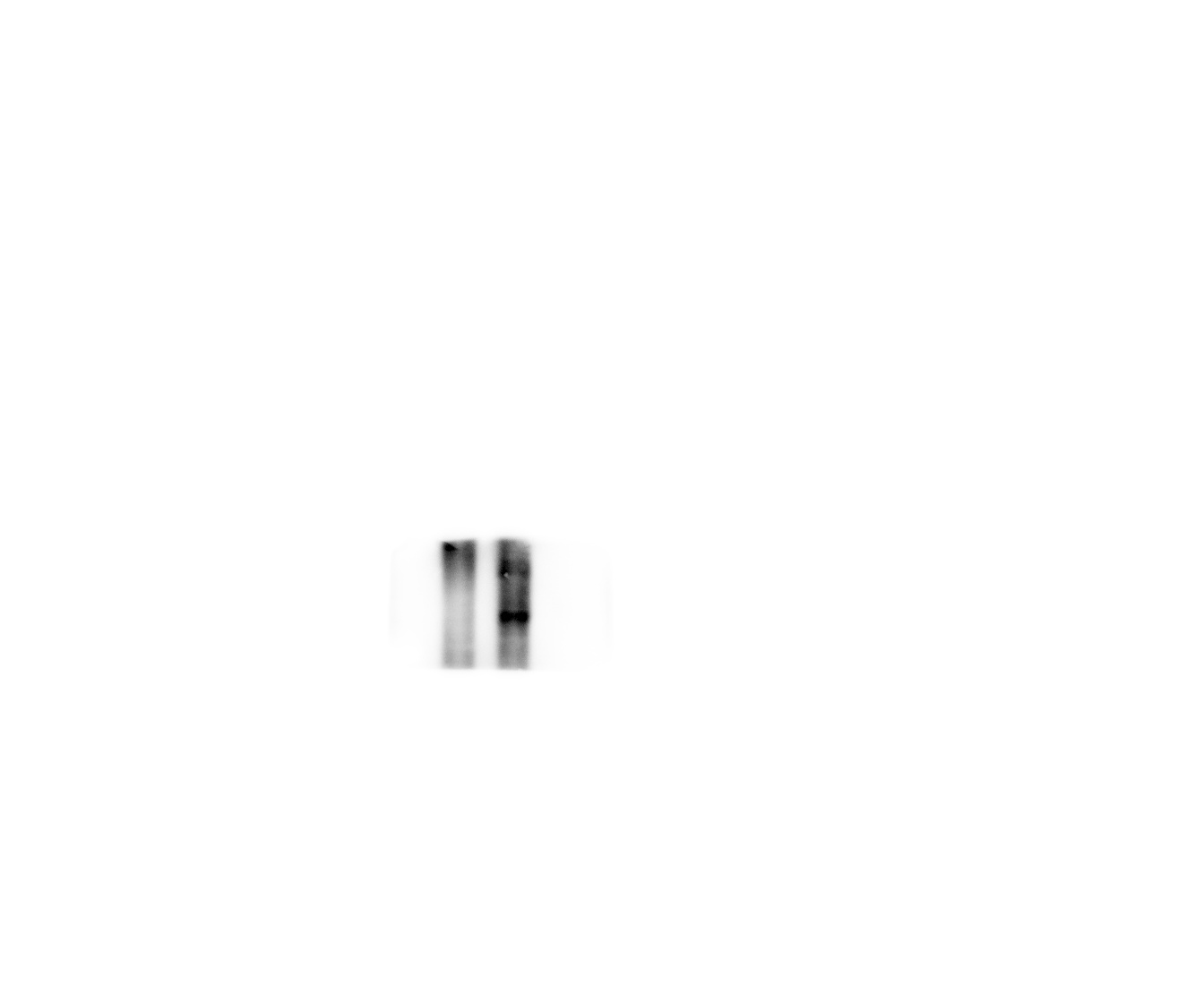

Supplement: Figure 2—source data 2. [file elife-103417-fig2-data2.zip › Figure 2-source data 2/IP-FOXP3 IB-CXXC1.jpg]

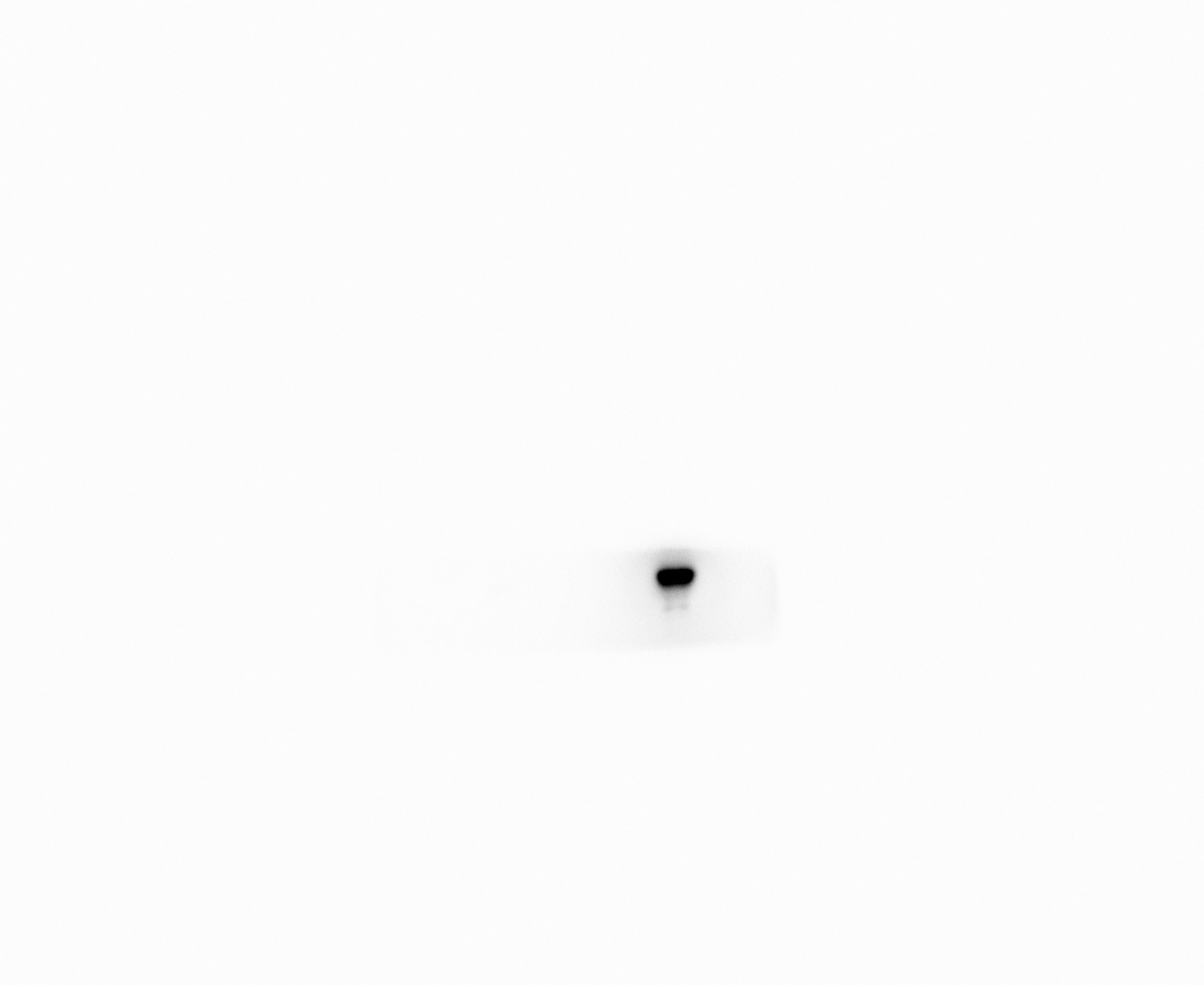

Supplement: Figure 2—source data 2. [file elife-103417-fig2-data2.zip › Figure 2-source data 2/IP-FOXP3 IB-FOXP3.jpg]

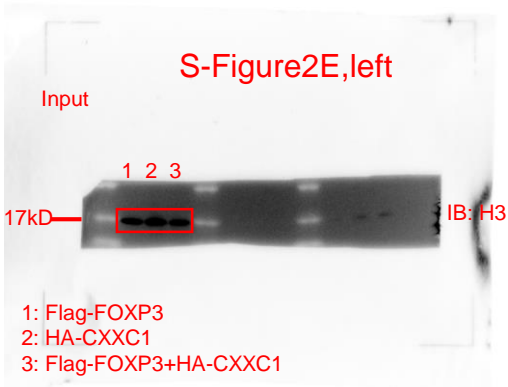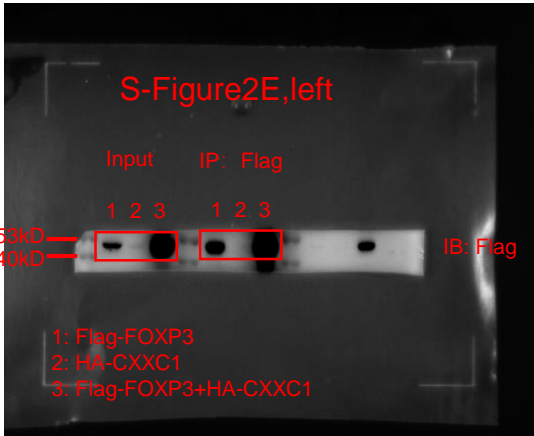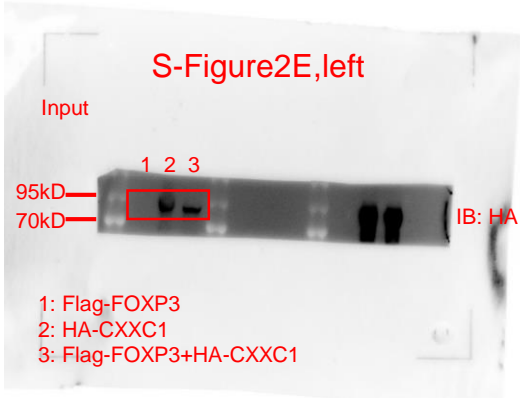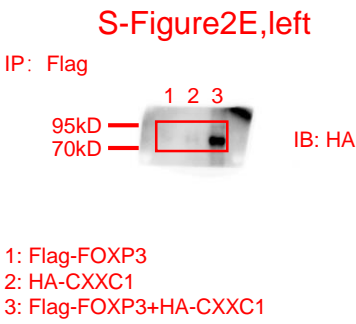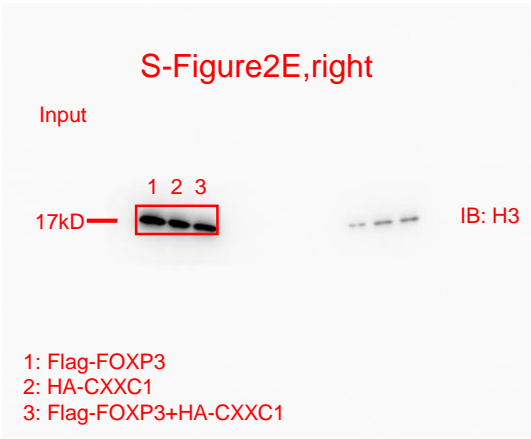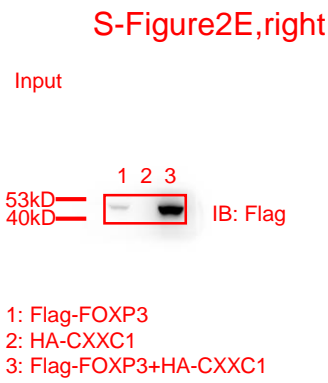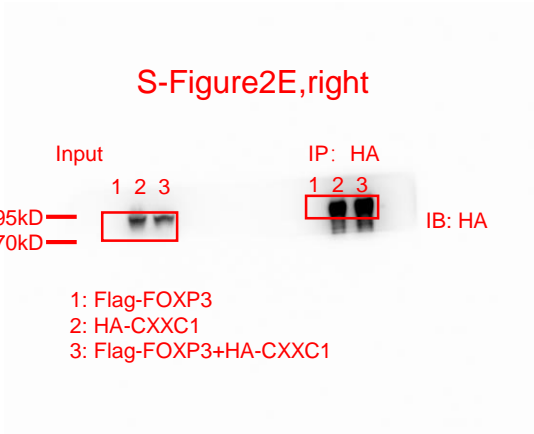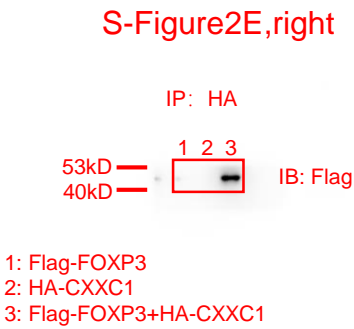

Supplement: Figure 2—figure supplement 1—source data 1. [file elife-103417-fig2-figsupp1-data1.zip › Figure 2-figure supplement 1-source data 1.pdf]

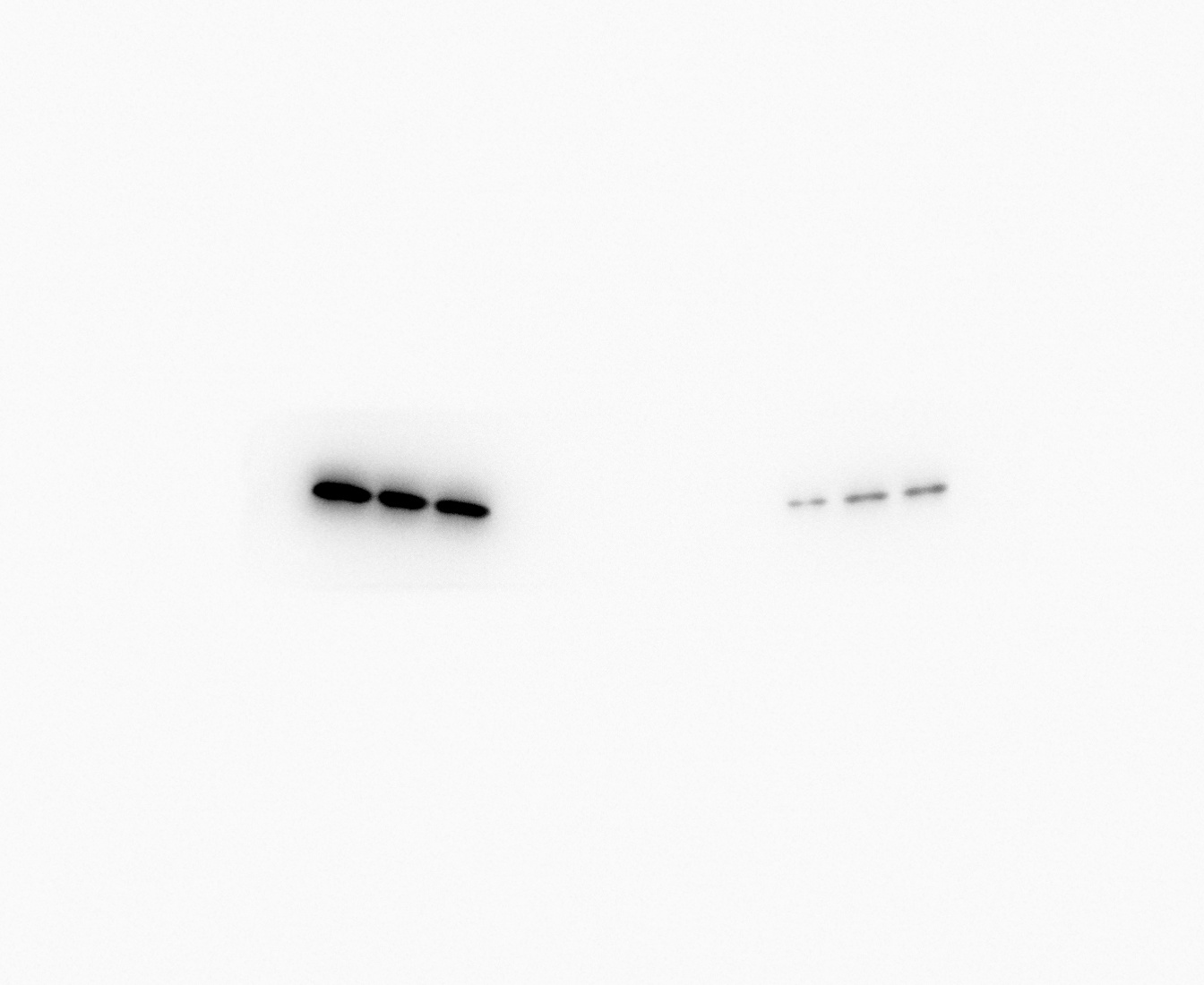

Supplement: Figure 2—figure supplement 1—source data 2. [file elife-103417-fig2-figsupp1-data2.zip › Figure 2-figure supplement 1-source data 2/H3.jpg]

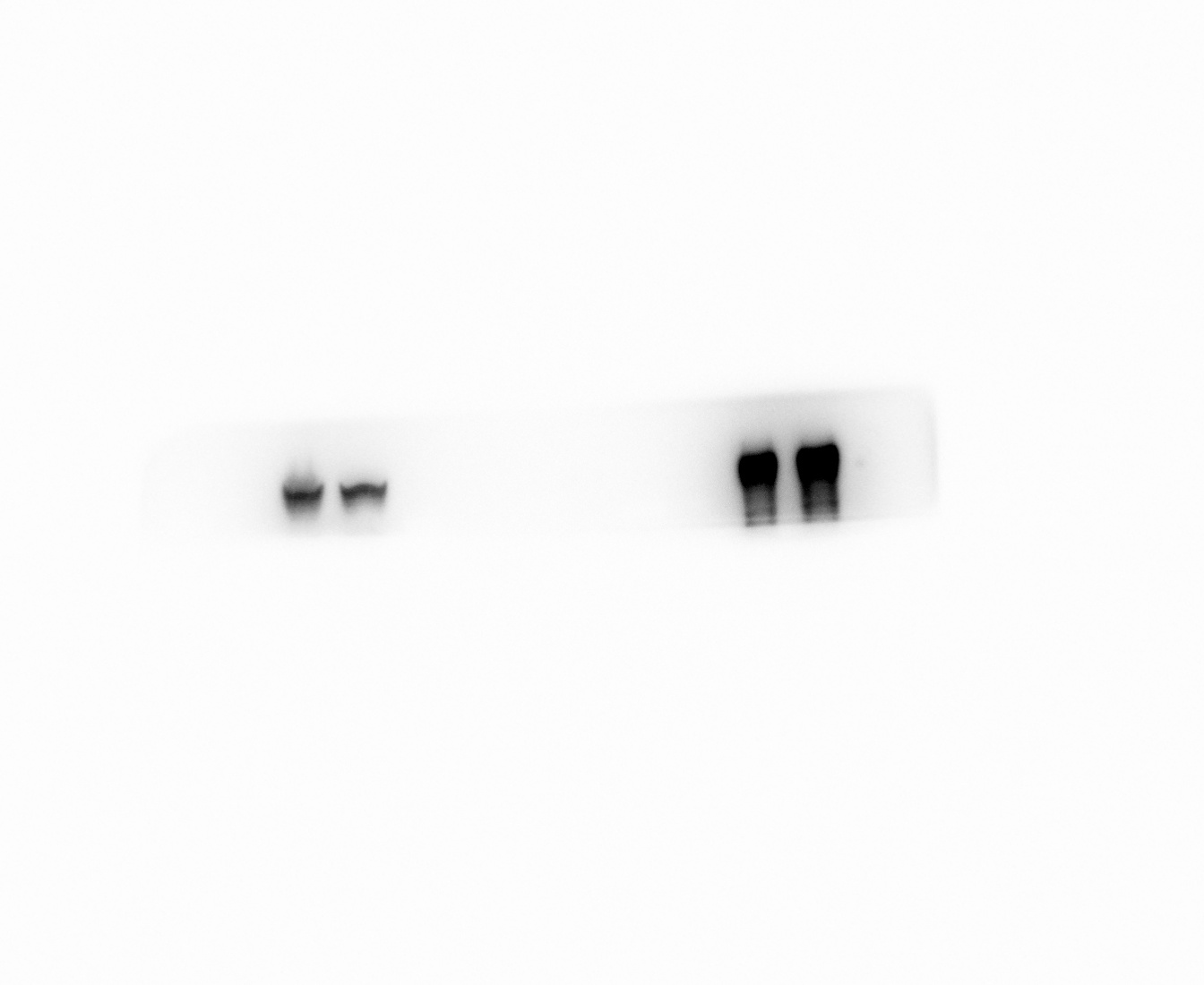

Supplement: Figure 2—figure supplement 1—source data 2. [file elife-103417-fig2-figsupp1-data2.zip › Figure 2-figure supplement 1-source data 2/Input IB-HA.jpg]

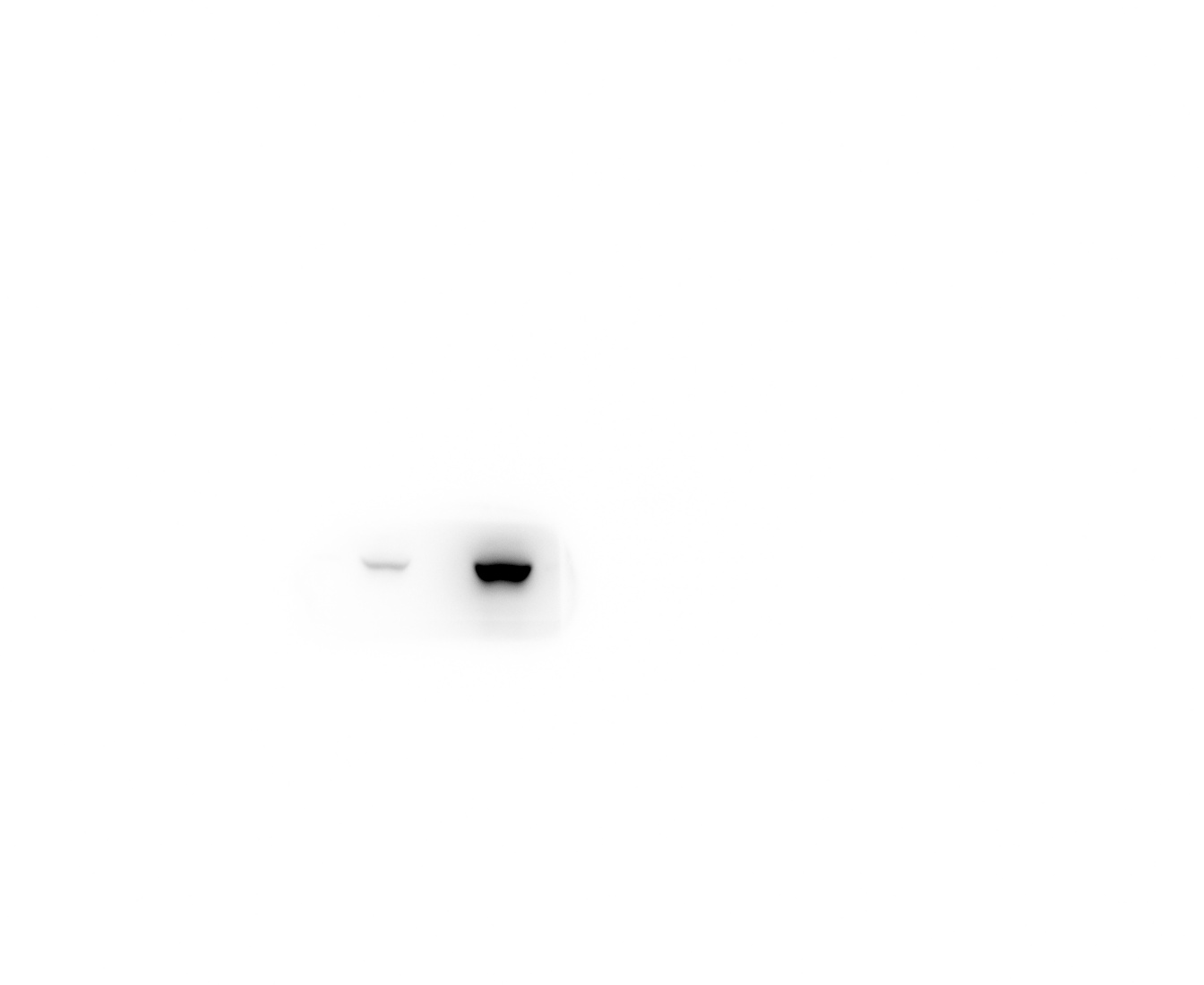

Supplement: Figure 2—figure supplement 1—source data 2. [file elife-103417-fig2-figsupp1-data2.zip › Figure 2-figure supplement 1-source data 2/Input IB-Flag.jpg]

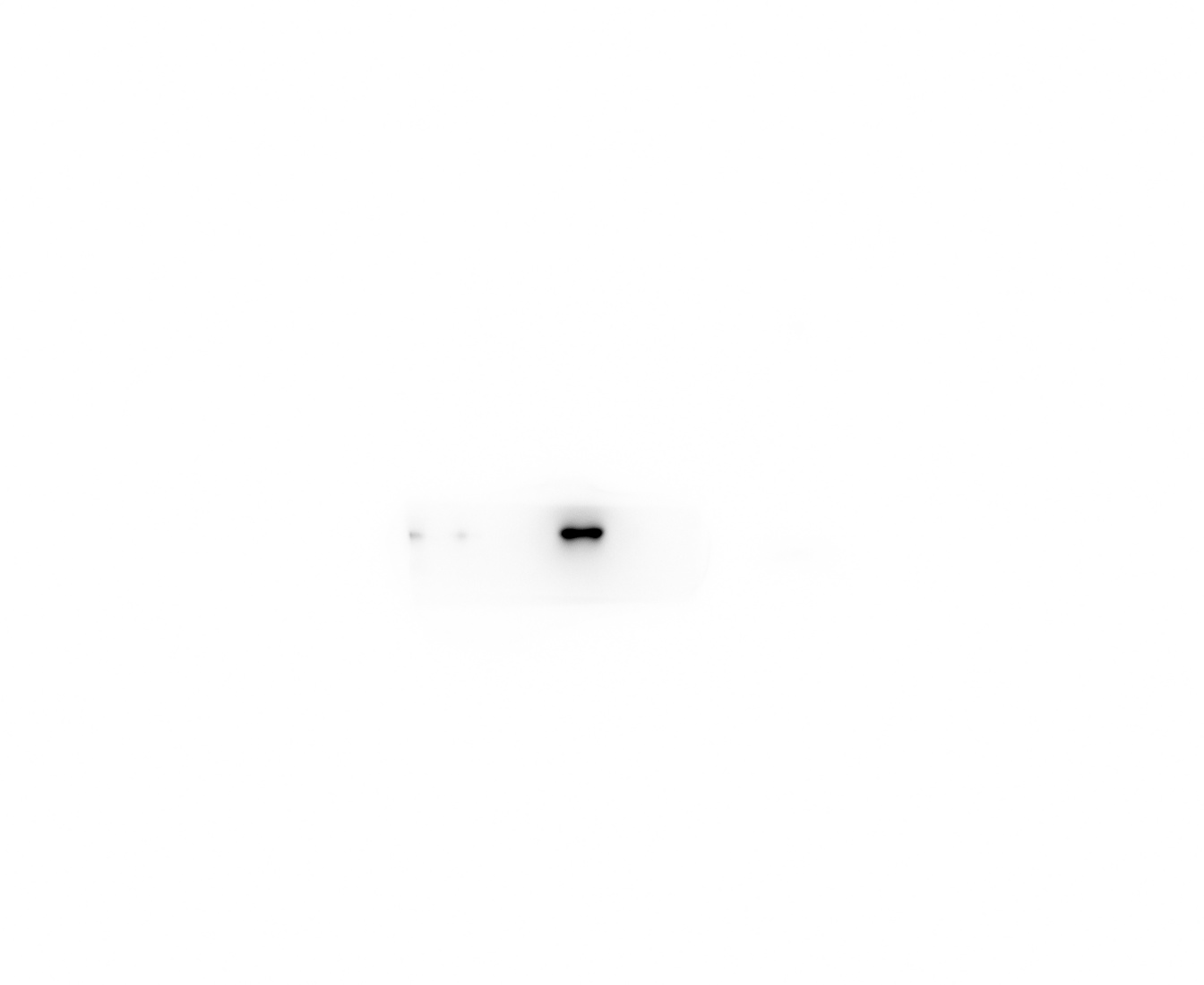

Supplement: Figure 2—figure supplement 1—source data 2. [file elife-103417-fig2-figsupp1-data2.zip › Figure 2-figure supplement 1-source data 2/IP-HA IB-FLAG.jpg]

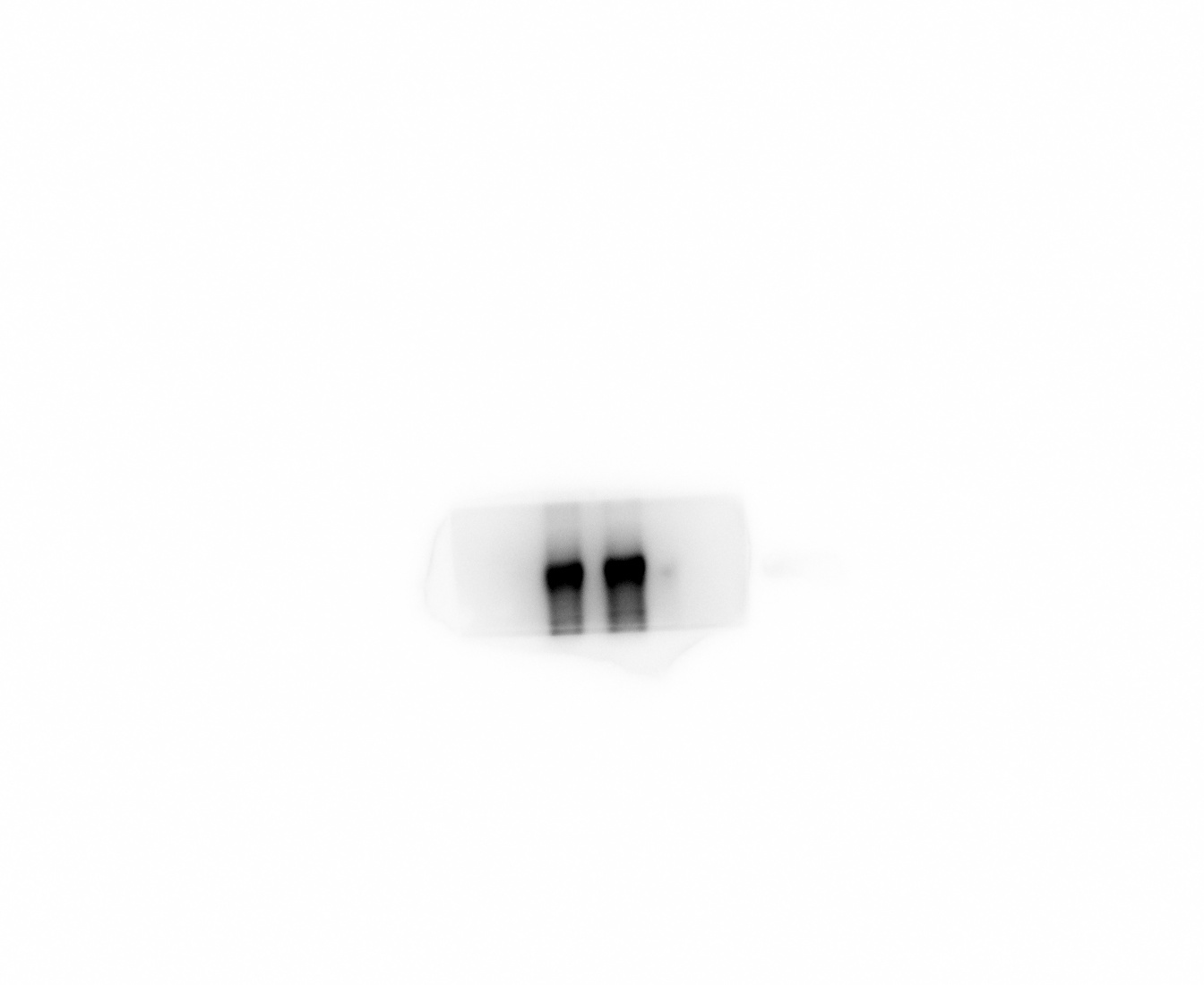

Supplement: Figure 2—figure supplement 1—source data 2. [file elife-103417-fig2-figsupp1-data2.zip › Figure 2-figure supplement 1-source data 2/IP-HA IB-HA.jpg]

S-Figure3A

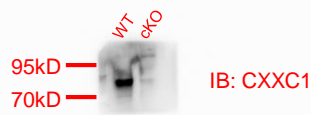

S-Figure3A

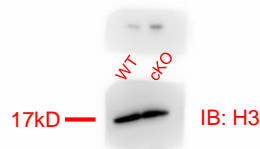

Supplement: Figure 3—figure supplement 1—source data 1. [file elife-103417-fig3-figsupp1-data1.zip › Figure 3-figure supplement 1-source data 1.pdf]

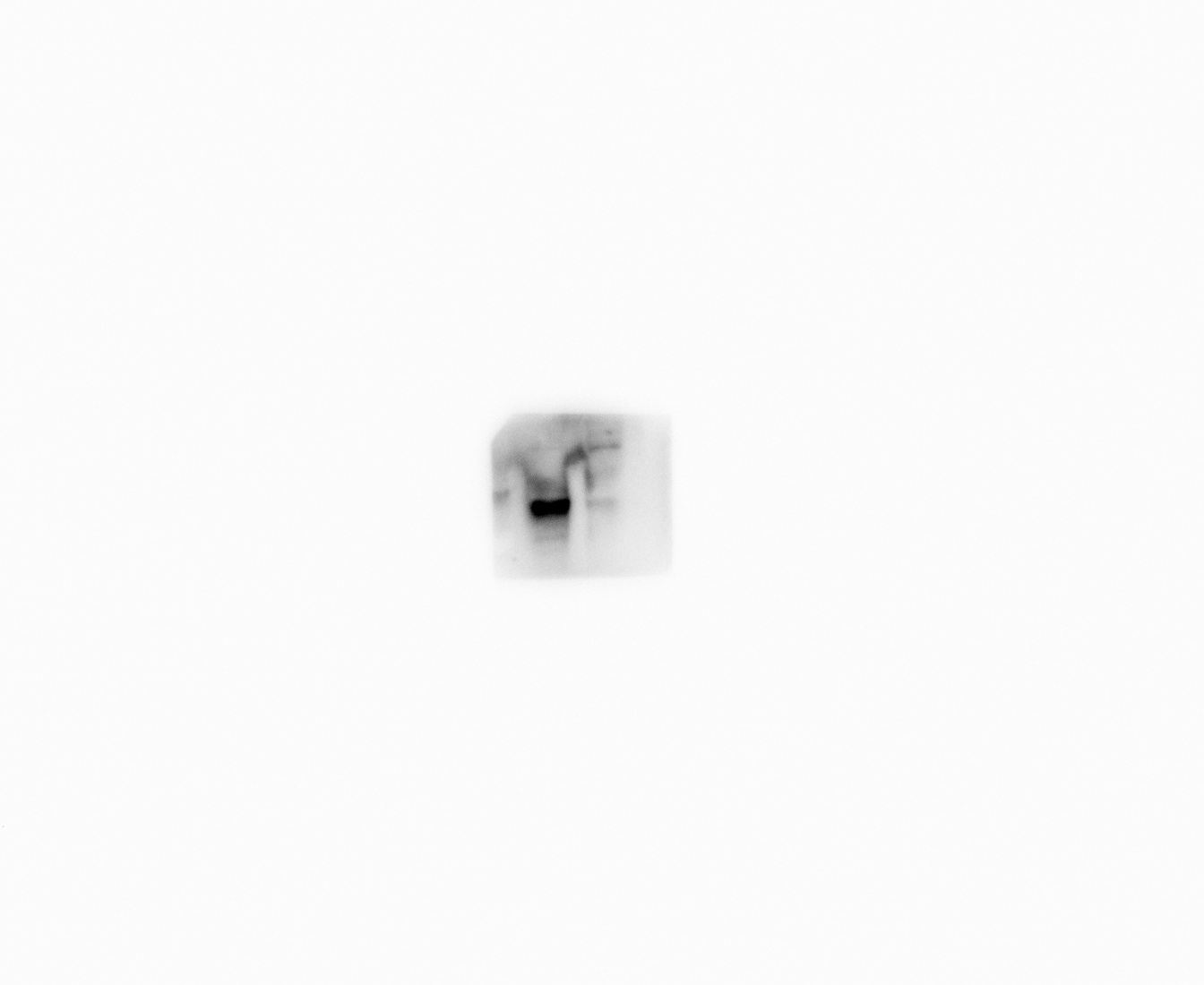

Supplement: Figure 3—figure supplement 1—source data 2. [file elife-103417-fig3-figsupp1-data2.zip › Figure 3-figure supplement 1-source data 2/CXXC1.jpg]

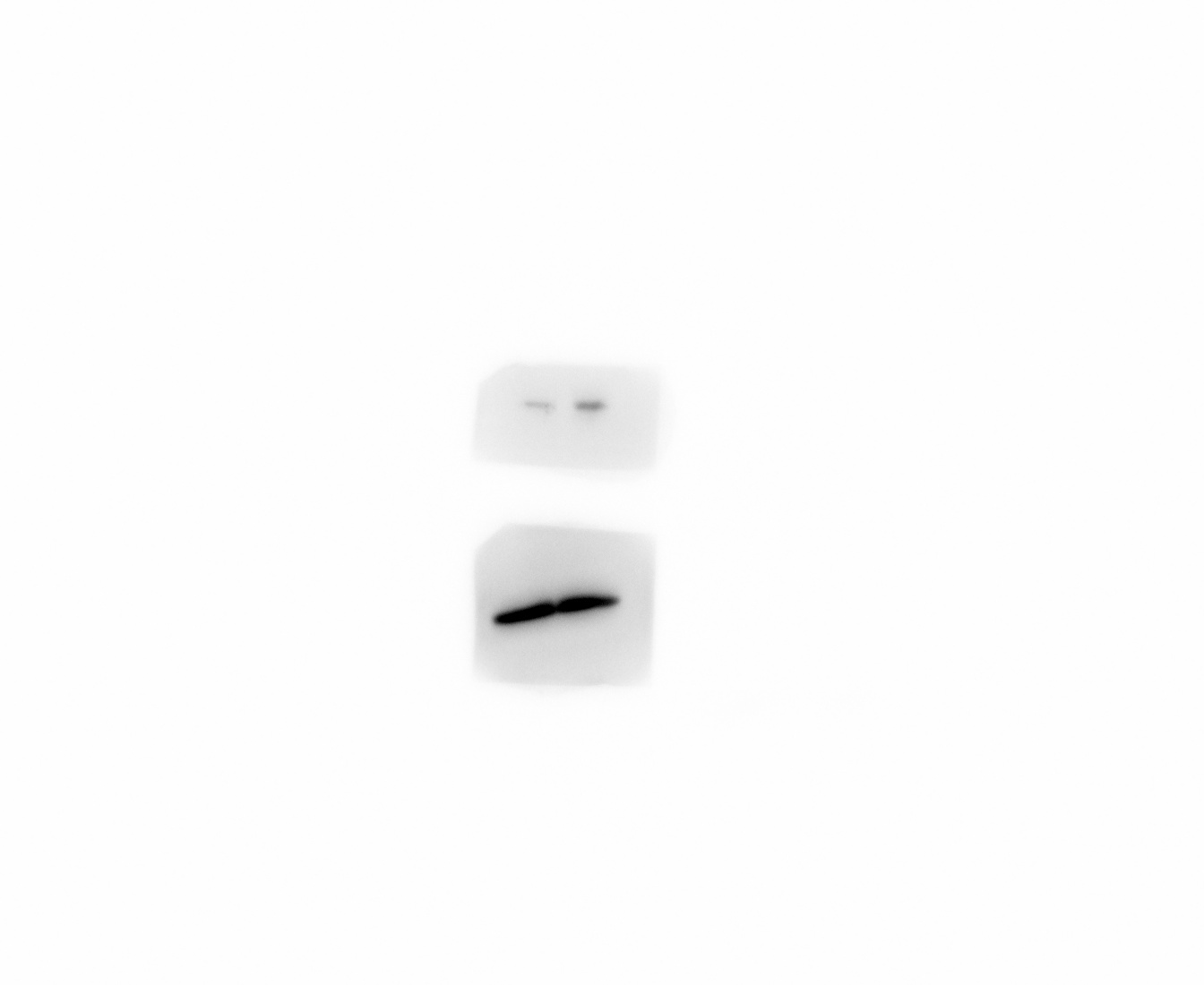

Supplement: Figure 3—figure supplement 1—source data 2. [file elife-103417-fig3-figsupp1-data2.zip › Figure 3-figure supplement 1-source data 2/H3.jpg]
