# Supplementary material for: CXXC-finger protein 1 associates with FOXP3 to stabilize homeostasis and suppressive functions of regulatory T cells
Source: eLife. 2025 Apr 4;13:RP103417. doi: 10.7554/eLife.103417 (PMC11970909; doi:10.7554/eLife.103417)
Supplement: Supplementary file 1. [file elife-103417-supp1.docx]

**supplementary file 1a**

| Sample | Total reads | Mapping efficiency | Correlation |
| --- | --- | --- | --- |
| WT-IgG | 28,433,877 | 97.42% |  |
| WT-CXXC1-rep1 | 37,605,589 | 98.00% | 0.96 |
| WT-CXXC1-rep2 | 40,625,734 | 97.64% |  |
| WT-H3K4me1-rep1 | 28,324,337 | 98.61% | 0.99 |
| WT-H3K4me1-rep2 | 23,814,172 | 99.20% |  |
| cKO-H3K4me1-rep1 | 26,325,592 | 98.74% | 0.97 |
| cKO-H3K4me1-rep2 | 22,378,739 | 99.20% |  |
| WT-H3K4me3-rep1 | 14,464,350 | 97.01% | 0.92 |
| WT-H3K4me3-rep2 | 41,233,666 | 99.06% |  |
| cKO-H3K4me3-rep1 | 10,423,744 | 97.86% | 0.88 |
| cKO-H3K4me3-rep2 | 22,365,710 | 98.88% |  |

**supplementary file 1b**

| Sample | Total reads | Uniquely Mapping efficiency |
| --- | --- | --- |
| Het-WT-rep1 | 35,360,089 | 92.9% |
| Het-WT-rep2 | 28,485,684 | 92.8% |
| Het-KO-rep1 | 34,522,848 | 93.0% |
| Het-KO-rep2 | 35,132,742 | 92.9% |

**supplementary file 1c**

| Sample | Total reads | Mapping efficiency | CpG methylation level |
| --- | --- | --- | --- |
| WT -WGBS | 461,596,431 | 77.0% | 79.4% |
| cKO -WGBS | 459,215,408 | 72.8% | 80.3% |
